# Effect of Kami Guibi-tang (KGT) in elderly subjects with insomnia: a study protocol from a single center, randomized, double-blind, placebo-controlled trial

**DOI:** 10.1186/s12906-023-04199-y

**Published:** 2023-10-23

**Authors:** Kyeong-Hwa Lee, Han-Gyul Lee, Seungwon Kwon, Seong-Uk Park, Woo-Sang Jung, Sang-Kwan Moon, Jung-Mi Park, Chang-Nam Ko, Seung-Yeon Cho

**Affiliations:** 1 Department of Clinical Korean Medicine, Graduate School, Kyung Hee University, Seoul, Republic of Korea; 2 Department of Cardiology and Neurology, Kyung Hee University College of Korean Medicine, Kyung Hee University Medical Center, Seoul, Republic of Korea; 3 Stroke and Neurological Disorders Center, Kyung Hee University College of Korean Medicine, Kyung Hee University Hospital at Gangdong, Seoul, Republic of Korea

**Keywords:** Insomnia, Subjective cognitive decline, Kami Guibi-tang, Herbal medicine, Randomized controlled trial, Protocol

## Abstract

**Background:**

The incidence of insomnia increases with age and is related to cognitive function in older adults; therefore, it is important to manage it actively. In this study, we report a protocol for the evaluation of the efficacy and safety of Kami Guibi-tang (KGT), a herbal prescription that has been widely used in East Asia for insomnia, forgetfulness, and depression, in older adults with insomnia.

**Methods:**

In this single-center, double-blind, randomized controlled trial, 60 older adults with insomnia and subjective cognitive decline will be recruited and randomly assigned to the KGT or placebo group. The KGT group will take KGT granules thrice a day for 12 weeks, whereas the control group will take placebo granules in the same manner.

Participants will be assessed for sleep, cognitive function, quality of life, and depression using the Pittsburgh Sleep Quality Index-Korean (PSQI-K), Insomnia Severity Index-Korean (ISI-K), Seoul Neuropsychological Screening Battery–Dement (SNSB-D), 36-item MOS Short Form Survey (SF-36) and Short version of the Geriatric Depression Scale (S-GDS) before and at the end of administration of the investigational product. The PSQI-K, ISI-K, and SF-36 will be further assessed 12 weeks after the end of medication to determine whether the effects on sleep and quality of life are sustained. The PSQI-K total score difference between the two groups at 12 and 24 weeks will be the primary outcome; all other endpoints will be secondary.

Safety will be assessed by performing blood tests and electrocardiograms before taking the investigational drug, 6 weeks after taking the drug, and 12 weeks after taking the drug; any adverse events will be observed throughout the study.

**Discussion:**

The protocol will provide a detailed process for a clinical trial to evaluate the efficacy and safety of KGT in elderly patients with insomnia. We will also investigate if changes in cognitive function correlated with improvements in insomnia.

**Trial registration:**

This trial was registered at CRIS (Clinical Research Information Service) on April 27, 2023 (KCT0008391, version 2.0). https://cris.nih.go.kr/cris/search/detailSearch.do?seq=24811&search_page=L.

**Supplementary Information:**

The online version contains supplementary material available at 10.1186/s12906-023-04199-y.

## Background

Insomnia disorder is characterized by complaints of one or more of the following symptoms: difficulty initiating sleep, difficulty maintaining sleep, or early morning awakening with an inability to return to sleep, at least 3 nights per week for at least 3 months by the Diagnostic and Statistical Manual of Mental Disorders, Fifth Edition (DSM-5) [1]. Insomnia is a common symptom observed in clinical practice, and approximately 30–50% of the total population experiences intermittent short-term insomnia [2]. Insomnia is more commonly observed in women than in men and increases with age [3]. Sleep deprivation is a risk factor for cardiovascular diseases, including myocardial infarction, coronary artery disease, heart failure, and stroke, and is estimated to increase cardiovascular mortality [4–6]. Additionally, insomnia has been associated with neurocognitive function [7], processing speed, attention conversion, and short-term visual memory deterioration when sleep time is short [8].

In clinical practice, sleeping pills are often initially prescribed for insomnia, and while they relieve patients’ sleep deprivation effectively in a short period, they can also cause problems of abuse and dependence [9]. In some patients, the use of sleeping pills is accompanied by adverse effects, withdrawal symptoms, and rebound insomnia. Moreover, the effects of long-term use may not be clear, as the risk of side effects increases [10]. Particularly, when elderly patients use sleeping pills for a long time, side effects such as cognitive impairment, daytime fatigue, and increased risk of falls have been reported [11].

While insomnia can cause cognitive decline [12], frequent use of sleep medication is also associated with an increased risk of dementia in some older adults [13, 14]. Therefore, in addition to conventional treatments, it is necessary to develop other effective and safe treatments for long-term use in elderly patients with insomnia. Herbal medicines that use natural products should be considered.

Kami guibi-tang (KGT; Kami-guibi-tang in Korean, Jia-wei-gui-pi-tang in Chinese, and Kami-kihi-to in Japanese) is a herbal medicine widely used for insomnia, cognitive decline, and depression [15]. KGT improved sleep defects in Parkinson’s disease models [16]. In clinical studies, it has been reported to be effective in preventing sleep deepening in patients with menopausal symptoms after treatment for gynecological malignancy [17] and in patients with cancer-related sleep disturbance [18]. Additionally, the mechanisms and effects related on cognitive function have been reported in vivo and in clinical studies [19–22].

However, few clinical studies have reported on the efficacy and safety of KGT in elderly patients with insomnia. In particular, since it has already been shown that insomnia and cognitive decline are related, there is a need to observe both symptoms in the elderly [23]. The primary aim of this clinical trial is to confirm the efficacy and safety of KGT in elderly patients with insomnia. Second, we aimed to explore whether KGT was effective in improving subjective cognitive decline in elderly individuals.

## Methods and analysis

### Study design

**Fig. 1:**
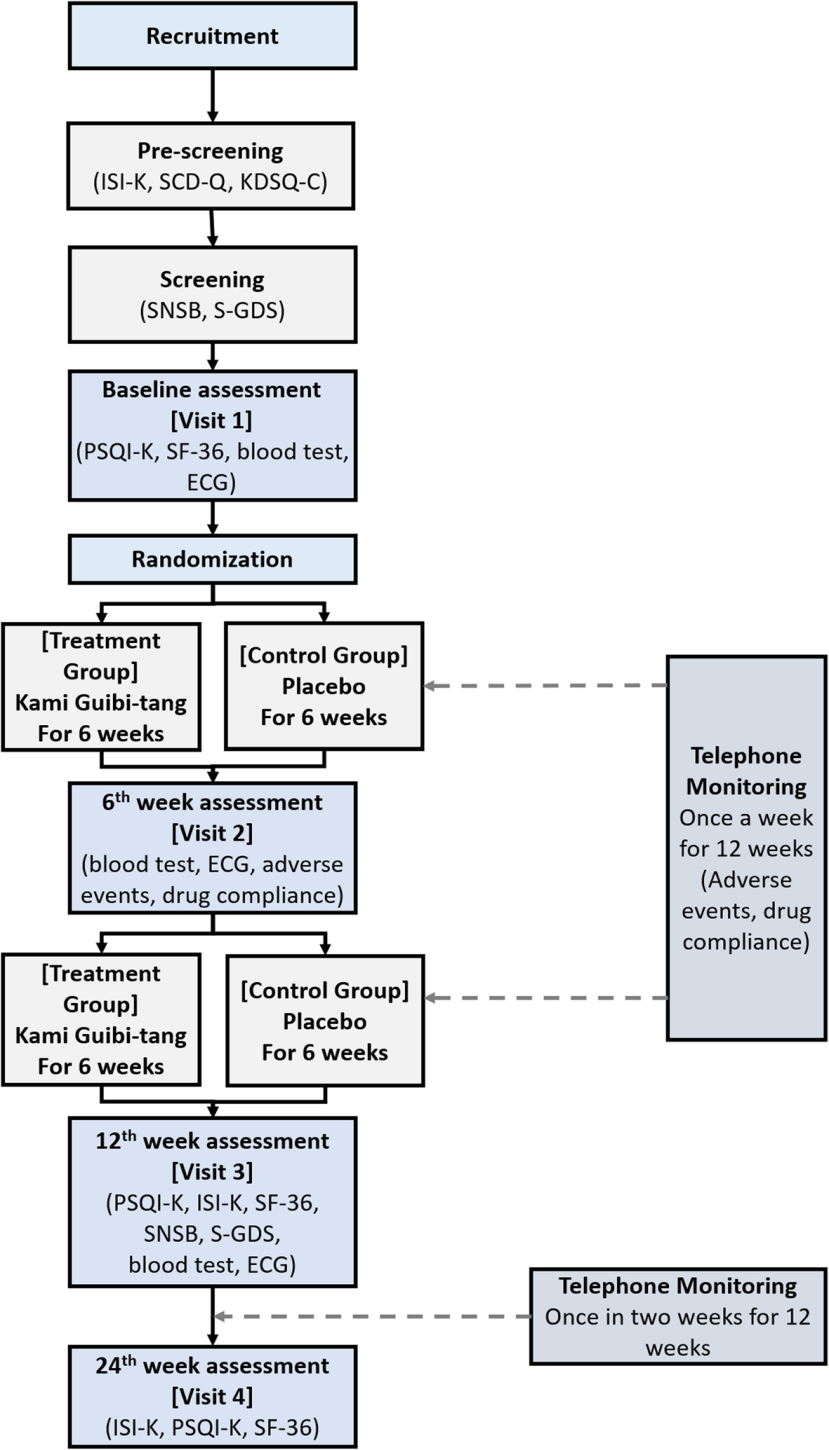
Flowchart of the study process

 This single-center, randomized, double-blind, placebo-controlled clinical trial aims to analyze the efficacy and safety of KGT granules in elderly patients with insomnia. Participants with insomnia will be administered KGT or placebo granules, one pack at a time, three times a day for 12 weeks. The effect of insomnia improvement will be evaluated using the Pittsburgh Sleep Quality Index-Korean (PSQI-K) [24] and the Insomnia Severity Index-Korean (ISI-K). The primary efficacy endpoint is the PSQI-K score, and we will assess the change in the total score at baseline, after 12-weeks and then 12 weeks after the end of the dosing period. The ISI-K will be evaluated with the same time as PSQI-K. We will evaluate changes in cognitive function using the Seoul Neuropsychological Screening Battery (SNSB) [25, 26], quality of life using the 36-item MOS Short Form Survey (SF-36) [27], and depression using the Korean version of the Short-Form Geriatric Depression Scale (SGDS-K) [28]. The enrolment, interventions, and assessments of the study are summarized in Table 1. A schematic of the study process is shown in Fig. 1. This protocol was reported in accordance with the Standard Protocol Items: Recommendations for Interventional Trials (SPIRIT) guidelines (Additional file 1).

**Table 1:**
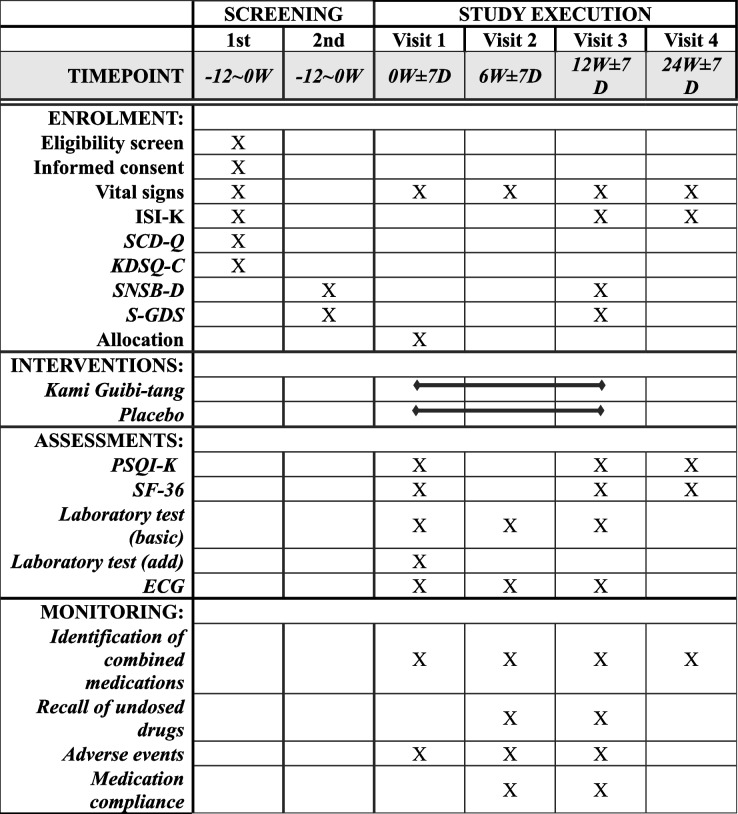
The schedule of enrolment, interventions, and assessments

Laboratory test (basic): AST, ALT, glucose, BUN, creatinine, Na, K, Cl, LDH, CPK

Laboratory test (add): Cholesterol, HDL-cholesterol, LDL-cholesterol, triglyceride, homocysteine, T3, free T4, TSH

ECG: electrocardiogram

*ISI-K* Insomnia Severity Index-Korean, *SCD-Q* Subjective Cognitive Decline Questionnaire, *KDSQ-C* Korean Dementia Screening Questionnaire-Cognition, *SNSB-D* Seoul Neuropsychological Screening Battery-Dementia version, S-GDS = Korean version of Short form Geriatric Depression Scale, *PSQI-K* Pittsburgh Sleep Quality Index-Korean, *SF-36* 36-item MOS Short Form Survey

### Study setting

The Kyung Hee University Hospital at Gangdong, Seoul, the Republic of Korea, is the site of the study. Recruitment will be conducted from May 2023 to October 2024 through hospital bulletin boards, newspapers, and online advertisements.

### Eligibility

#### Inclusion criteria

Participants who meet all the criteria below are eligible to participate:

1. Those who complain of insomnia according to the criteria presented by DSM-5 > 65 years.
2. Those with an Insomnia Severity Index Korean (ISI-K) score of > 8.
3. Those with a Subjective Cognitive Decline Questionnaire (SCD-Q) score of > 7 and a Korean Dementia Screening Questionnaire-Cognition (KDSQ-C) score of < 6.
4. Those who have not received any new treatments related to insomnia within the last 2 weeks: pharmacological and non-pharmacological treatments.
5. Those who have not received cognitive-related drug treatments within the last 2 weeks.
  Cognitive-related drugs include cerebral function-improving agents, cerebral circulation-activating drugs, and other drugs or nutrients that can affect cognition.
6. Those who have been in a stable state without any change in medications for underlying diseases for the past 4 weeks or more and who are planning to take stable medications during the study period.
7. Those who have no problems with communication.
8. Those who voluntarily decided to participate and signed the written consent form.

#### Exclusion criteria

The exclusion criteria are as follows:

1. Diagnosis of dementia according to the criteria of the National Institute of Neurological and Communicative Disorders and Stroke and Alzheimer’s Disease and Related Disorders Association (NINCDS-ADRDA) [29].
2. Diagnosed with mild cognitive impairment according to the diagnostic criteria for mild cognitive impairment suggested by Albert et al. [30]
3. Patients with a brain disease that causes other neurological symptoms.
4. Patients with a mental disorder, a behavioral problem requiring psychotropic drug treatment, or those who are taking a psychotropic drug.
5. Patients with substance abuse (drug addiction, alcoholism, etc.)
6. Patients with a physical disability that threatens life and requires immediate treatment.
7. Uncontrolled hypertension patient.
8. Patients with heart or kidney disease requiring acute treatment.
9. Patients with edema.
10. Gastrointestinal symptoms (anorexia, stomach discomfort, nausea, abdominal pain, and diarrhea).
11. Those who are taking drugs that may cause hypokalemia or myopathy.
12. Patient with hypersensitivity to the components of investigational drug.
13. Clinically significant abnormalities in blood chemistry tests (AST/ALT: more than 2 times the upper limit of normal; serum creatinine: more than 10% of the upper limit of normal).
14. Patient who participated in another clinical study and received intervention within 4 weeks.
15. Illiteracy.
16. Others determined by the researcher to be inappropriate.

#### Drop-out criteria

The investigators will discontinue the administration of the investigational drug and drop the subjects according to the dropout criteria. The subjects can also voluntarily withdraw from the trial at any time. The criteria for dropout are as follows:

1. When a serious adverse event occurs or when the participant requests discontinuation of the study due to an adverse event.
2. When a systemic disease is found that was not detected in the examination prior to the administration of the investigational drug.
3. In case the participant or the participant’s legal representative requests to discontinue the study.
4. The medication compliance is < 60% at visit 2 (6 weeks after taking the investigational drug).
5. The medication compliance is < 80% at visit 3 (12 weeks after taking the investigational drug).
6. When the participant withdraws consent to participate in the clinical trial.
7. In case the participant is not tracked.
8. In the case of taking drugs that may affect the study results without the investigators’ instruction or consent during the study period.
9. In other cases where the investigators determine that it is inappropriate to proceed with the study.

### Recruitment

Sixty participants will be enrolled through hospital bulletin boards, newspapers, and other online advertisements. Those who wish to participate in the study will be enrolled as subjects, after they have gone through the screening process and after obtaining written consent. After the investigator explains the contents of the informed consent form to the participants and ensures that they understand the contents, participants can grant their permission to participate in the study of their own free will. The screening items are as follows: demographic information, medical history, onset time and pattern of insomnia, vital signs, Insomnia Severity Index-Korean (ISI-K), Subjective Cognitive Decline Questionnaire (SCD-Q), Korean Dementia Screening Questionnaire-Cognition (KDSQ-C), and Seoul Neuropsychological Screening Battery (SNSB-D). The SNSB-D includes a Short version of the Geriatric Depression Scale (S-GDS).

### Randomization, allocation concealment, and blinding

Sixty participants who meet the criteria will be assigned to the treatment or control group at a ratio of 1:1 by an investigator independent of this clinical study using the block randomization method. A random assignment table is randomly assigned according to a random number table generated using the RAND function in Excel. According to the randomization table, the investigational drug manufacturer packs the treatment drug and placebo in the same manner, labels the serial number in the form of R-00, and provides it to the principal investigator. Registered pharmacists manage the investigational drugs and provide them to the participants according to their randomization number.

To keep the participants blind, the authenticity of the drug will not be disclosed until the end of the study period. The properties of the placebo are the same as those of the treatment drug, as to maintain double blindness. The investigators, coordinators, and pharmacists should remain blind to the assignments until the end of the study period.

### Intervention

KGT or placebo granules (one dose: 3.0 g) are orally administered with water 3 times a day (30 min after meals) for 12 weeks. KGT is a herbal medicine approved by the Ministry of Food and Drug Safety and is manufactured by Kyoung Bang Pharmaceutical Co., Ltd. (Incheon, Republic of Korea). It is yellow-brown granules and consists of *Astragali Radix* 1.0 g, *Ginseng Radix* 1.0 g, *Atratylodis Rhizoma* 1.0 g, *Poria* 1.0 g, *Zizyphi Fructus* 0.67 g, *Zingiberis Rhizoma* 0.33 g, *Saussureae Radix* 0.33 g, *Glycyrrhizae Radix* 0.33 g, *Zizyphi Spinosi Semen* 1.0 g, *Longan Arillus* 1.0 g, *Angelicae Radix* 0.67 g, *Polygalae Radix* 0.67 g, *Bupleuri Radix* 1.0 g, *Moutan Radicis Cortex* 0.67 g, and *Gardeniae Fructus* 0.67 g. The placebo was manufactured at the National Institute for Korean Medicine Development (NIKOM) Herbal Medicine Production Center (GMP) in Daegu, Republic of Korea, with an appearance, taste, and smell similar to KGT.

The participants will be trained to take the drug three times a day and then return the remaining medication at their next visit. Regarding compliance with the investigational drug, the remaining drug brought in by the participant at the time of the next visit is identified and recorded on a case report form. Medication compliance (%) signifies that the participant’s actual dose is divided by the number of investigational drugs taken by the date of the participant’s visit based on the date of the last visit × 100. The actual dose is the number of returned drugs on the day of the participant’s visit, subtracted from the drugs provided to the participant. When visiting 6 weeks after taking the drugs, the investigator retrieves the unused dose and reversely check the total dose. If medication compliance is less than 60% at the 6th week of taking, the subject will drop out. Even if all doses were administered over the next 6 weeks, the final medication compliance is < 80%. The final medication compliance must be 80% or higher; if medication compliance is less than 80%, the participant is excluded from the per-protocol (PP) analysis group.

### Outcome measurements

The PSQI-K and ISI-K are evaluated to confirm the effect of KGT granules on insomnia in the elderly. This will be measured three times: before taking the investigational drug, at the end of 12 weeks of medication, and after 12 weeks of 12-week medication.

To evaluate cognitive function, the SNSB-D is measured twice: at screening and at the end of 12 weeks of medication. In addition, to evaluate quality of life, SF-36 is measured three times: before taking the investigational drug, at the end of 12 weeks of medication, and after 12 weeks of the 12-week medication. The S-GDS, which evaluates depression, is measured twice in total: before taking the investigational drug and at the end of 12 weeks of medication.

### Primary outcome

#### PSQI-K

The Pittsburgh Sleep Quality Index (PSQI) is a subjective evaluation tool for sleep quality that includes quantity, depth, and peace of sleep. Subjective sleep quality, sleep latency, sleep duration, sleep efficiency, sleep disturbance, sleep medication use, and daytime dysfunction are evaluated. Each domain has a minimum score of 0–3 points, and the total score of the seven subdomains ranges from 0 to 21 points [31]. The higher the total score, the higher the severity of the sleep disorder; if it exceeds 5 points, it is judged as a sleep disorder. In this study, the Korean version will be used that has been independently confirmed for validity and reliability [24].

The primary outcome is the difference between the treatment and placebo groups in terms of changes in the total PSQI-K score observed before taking the investigational drug, at the end of 12 weeks of medication, and after 12 weeks of 12-week medication.

### Secondary outcomes

#### PSQI-K

Changes in the total PSQI-K score observed before taking the investigational drug, at the end of 12 weeks of medication, and after 12 weeks of the 12-week medication in each group.

#### ISI-K

The insomnia severity index developed by Bastien et al. [32] was validated using the Korean version by Cho et al. [33]. On a scale of 0 to 4, the intensity of insomnia experienced in the previous two weeks, contentment with present sleep patterns, disruption of daily activities, distress brought on by sleep issues, and impairment of quality of life are all assessed. A score of less than 15 is categorized as a risk factor for insomnia. The total score runs from 0 to 28, with 0 being the lowest and 28 being the highest. We evaluate the ISI-K score before taking the investigational drug, at the end of the 12 weeks of medication, and after 12 weeks of 12-week medication in each group.

#### SNSB-D

The Seoul Neuropsychological Screening Battery is a test developed to evaluate cognitive function, and it is a representative Korean-style clinical scale tool consisting of tests with relatively simple instructions, performance, and standardized studies so that even the elderly with a low social education period can easily access them [25]. The SNSB-D provides a Global Cognitive Function score that sums up only the test results that can be assigned to the subtests of the five cognitive domains of the SNSB. The total score is out of 300, with 17 points for attention, 27 points for language, 150 points for memory, 36 points for visuospatial function, and 70 points for frontal/executive function. This is tested before administration of the investigational drug and at the end of 12 weeks of medication.

#### SF-36

The 36-item MOS Short Form-36 is the most widely used universal health-related QoL scale, consisting of eight domains and 36 items [27]. The physical component summary includes the following: physical functioning, physical role, bodily pain, and general health. The mental component summary includes vitality, social functioning, role emotion, and mental health. This is evaluated before taking the investigational drug, at the end of 12 weeks of medication, and after 12 weeks of 12-week medication in each group.

#### S-GDS

The short-form Geriatric Depression Scale is a 30-item Geriatric Depression Scale (GDS) developed by Yesavage et al. [34] to evaluate the level of depression and anxiety and has a total of 15 questionnaire items. A score of ≥ 8 indicates a depressed state, while a higher score indicates a severely depressed state. In this study, the Korean version standardized by Bae et al. (2004) [28] will be used before taking the investigational drug and at the end of the 12 weeks of medication.

### Safety assessments and monitoring

Adverse events will be investigated during each visit. Subjects who took the investigational drug at least once are eligible for safety analysis. Any undesirable medical findings indicating symptoms not observed prior to the study are considered adverse events.

At every visit, blood pressure, pulse rate, and body temperature are investigated to determine the participants’ health status. Laboratory tests for glucose, aspartate aminotransferase (AST), alanine aminotransferase (ALT), blood urea nitrogen (BUN), creatinine, sodium, potassium, chloride, lactate dehydrogenase (LDH), creatine phosphokinase (CPK), and electrocardiography (ECG) are investigated before taking the investigational drug and after 6 and 12 weeks of medication. Participants with abnormal laboratory test results should be followed up. Additionally, total cholesterol, HDL cholesterol, LDL cholesterol, triglyceride, homocysteine, T3, free T4, and thyroid stimulating hormone (TSH) levels are investigated at baseline.

After visit 1 (baseline), the investigators monitor status of medication, adverse events, and concomitant medications via phone calls once a week until the next visit. After the end of the medication at visit 3 (12 weeks), the investigators monitor adverse events and concomitant medications via phone call every two weeks until the final visit.

### Concomitant medication

It can be allowed at the discretion of the investigators if it does not fall under the prohibited drug category among the drugs taken at least 2 weeks before participating in the study. Drugs used for the transient treatment of other diseases should be administered in consultation with the investigators. For all other drugs, information (product name, purpose of administration, duration, dosage, etc.) are recorded in a case report form.

Drugs and non-pharmacological treatments that may improve insomnia or affect cognitive function are prohibited. If the participants have taken these drugs or received treatment during the medication period, the study will be discontinued.

### Sample size

The herbal medicine, Kami Guibi-tang (KGT), used in this study is already approved for insomnia by the Korean Food and Drug Administration. This study aimed to evaluate insomnia in older adults and assess cognitive function. According to the central limit theorem, a sample consisting of > 30 individuals per group is assumed to be normally distributed; therefore, the sample size for this study was 30 individuals per group. If the normality test confirms that the distribution is normal, we will perform statistical analysis parametrically [35].

### Statistical analysis

All statistical analyses is a two-sided and the significance level is set at 5%. Statistical program SPSS (Ver 25.0 or more updated, IBM Corporation) will be used as the primary analysis tool. All participants randomized according to the intent-to-treat (ITT) analysis principle are considered as the Full Analysis Set (FAS). Participants who have completed taking investigational product for 12 weeks and completed up to the follow-up period (week 24) after 12 weeks are referred to as the protocol compliance analysis group, as per-protocol (PP).

#### Efficacy analysis

Efficacy evaluation is based on the FAS analysis group, and efficacy is analyzed and presented with reference to the PP analysis group. For safety evaluation, the ITT analysis group is the main assay.

1. Analysis of primary efficacy endpoints.

Participants with PSQI-K scores before the study and at least once after consuming the investigational product are analyzed. Based on the baseline, the difference between the two groups in the total PSQI-K score change at weeks 12 and 24 is analyzed using repeated measures analysis of variance (RM ANOVA) and linear mixed model (LMM) methods.

2) Analysis of secondary endpoints.

The PSQI-K score change will be analyzed by a paired t-test within each group for changes at 12 and 24 weeks based on the baseline. The ISI-K score change will be analyzed using the paired t-test within each group and the independent t-test between the two groups at the 12-week dosing period and at 24 weeks based on the baseline. The SNSB-D and S-GDS score changes will be analyzed using the paired t-test within each group and the independent t-test between the two groups with respect to the score change at week 12, based on the baseline. The SF-36 score change will be analyzed using a paired t-test within each group and an independent t-test between the two groups at 12 and 24 weeks based on the baseline. Changes over time will be analyzed using the RM ANOVA and LMM methods. The estimated values of the mean difference between the groups, 95% confidence intervals, and p-values will be presented.

#### Adverse events

An adverse event is a symptom that was not observed before the administration of the investigational drug, which is a new symptom during the dosing period. This is a generic term for temporary phenomena related to the administration of investigational drugs. Events (symptoms, onset date, duration, etc.) expected to be adverse events of the investigational drug should be recorded in the adverse event report. The severity of adverse events is evaluated according to the severity of symptoms, by referring to the evaluation criteria of the investigator. A degree that does not interfere with normal daily activities (functions) to such an extent that the participant hardly notices it or the degree to which most of them do not require treatment is evaluated as mild. If the participant feels discomfort to a degree that interferes with normal daily life (function) or the participant is deemed to continue the study but may require treatment, it is evaluated as moderate. The degree to which daily life (function) is impossible because the participant is very uncomfortable, or to a degree that continuous study participation is deemed impossible, such that treatment or hospitalization may be required, is evaluated as severe. The responsibility of providing medical care to the injured participant lies with the principal investigator.

## Discussion

Insomnia is a common symptom that can cause various physical and psychological problems [9], and it has been reported that one-third of the total American population suffers from sleep-associated complications [36]. A survey on the prevalence of insomnia symptoms in the elderly in Europe showed that the prevalence was variously investigated as 16.6% in Denmark and 32.1% in Poland [3]. A study using the National Health Insurance Service-National Sample Cohort (NHIS-NSC) from 2002 to 2013 in South Korea [37] reported that one in ten people over the age of 60 and one in five from the age of 80 years or older showed a significant increase in the number of patients with insomnia as they aged. The proportion of the population aged ≥ 65 years is expected to increase from 9.8% to 2022 to 20.1% in 2070 [38]; therefore, it is estimated that the number of elderly patients with insomnia will also increase.

Insomnia is more common in the elderly, as aging decreases deep sleep and leads to more frequent awakenings and a tendency to wake up earlier in the morning [39]. This leads to fatigue, disruption of daily activities, decreased quality of life, and increased depression [40, 41]. Additionally, sleep disorders can cause cognitive decline in older adults, worsening mild cognitive decline [42] or increasing the risk of Alzheimer’s disease (AD) [43]. Sleeping pills, a common treatment for older adults with insomnia, can cause cognitive decline. Older adults who reported taking sleeping pills had an increased risk of developing dementia 15 years later [13].

Seniors with sleep disorders experience discomfort owing to poor cognitive function, and the frequent use of sleeping pills to treat insomnia can also lead to cognitive decline. Therefore, there is a need for safe and effective treatments for older adults with insomnia without cognitive decline.

KGT is a traditional herbal prescription that has been widely used for insomnia, forgetfulness, and depression in East Asia. Therefore, it can be prescribed to older adults with insomnia who experience memory loss. A clinical study of patients with cancer with sleep disorders showed that KGT was effective [18]; however, no such studies have been reported in older patients with insomnia.

The memory-enhancing effects and mechanisms of KGT have been confirmed in several in vivo, in vitro, and clinical studies. KGT extended the survival period of mice injected with N-methyl-d-aspartic acid (NMDA) and showed a protective effect against delayed neuronal death in cornu ammonis 1 (CA1) pyramidal cells of the gerbil hippocampus [44]. In a study on age-induced learning performance impairment using senescence-accelerated mice (SAM), the retention rate in the step-through test increased, the number of errors in the step-down test decreased, and the conditioned avoidance response rate in the shuttle-box test increased in the group administered KGT. It has been objectively confirmed that KGT could improve learning ability in aging models, and the possibility of using KGT to treat cognitive decline due to aging has been suggested [45].

In a clinical study of 75 patients with AD in Japan, treatment with KGT for 3 months significantly improved the MMSE score compared to the control group, suggesting the potential for the clinical application of KGT in cognitive decline [46].

KGT also improved cognitive function in 30 patients with amnestic mild cognitive impairment (MCI) [19]. This preliminary study showed that 24 weeks of KGT administration in patients with amnestic MCI resulted in a significant improvement in the CDR-SB (Clinical Dementia Rating-Sum of Boxes) scores, whereas the scores worsened in the placebo group. Additionally, the total and memory domain scores of the Seoul Neuropsychological Battery (SNSB-D) were significantly improved in the KGT group compared to the placebo group, indicating that the overall cognitive function of the KGT group was significantly improved compared to the placebo group. This suggests the feasibility of using KGT in amnestic MCI patients with a high risk of progression to dementia.

Insomnia and cognitive decline are particularly important due to their negative effects such as reduced quality of life and high economic costs [47]. A meta-analysis showed that insomnia groups perform worse in various areas of cognitive functioning, such as working memory, episodic memory, and some executive functions [48], and chronic insomnia has been reported to cause atrophy of the CA1 subregion, leading to cognitive impairment [49]. After all, lack of sleep can affect future cognitive function; therefore, getting adequate sleep is crucial for maintaining cognitive function in older adults.

Elderly patients with insomnia experience cognitive decline due to insomnia; therefore, it is necessary to study whether prescriptions traditionally used for insomnia are effective in the elderly, considering their characteristics, such as cognitive decline.

We hypothesized that KGT would be effective and safe for improving sleep status, cognitive function, and quality of life in elderly individuals with insomnia and subjective cognitive decline. This clinical study will generate data suggesting the therapeutic efficacy of KGT in insomnia in older adults. If the results of this study prove the efficacy and safety of KGT, it will serve as a basis for further use in the elderly with insomnia and will also contribute to the prevention of memory decline due to insomnia in the long term.

The limitation of the study design is that the inclusion criteria included not only those who do not take sleeping pills but also those who take sleeping pills if there was no change in their medication. The study period can be extended if the recruitment period is prolonged, and any changes to the study, including termination, will be made after approval from the IRB.

### Trial status

Recruitment of participants is currently in progress. Eight subjects were enrolled on Aug. 25, 2023.

### Supplementary Information

**Additional file 1.** SPIRIT 2013 checklist.

## Data Availability

Not applicable; no data have yet been generated.

## Abbreviations

ALT: Alanine aminotransferase
AST: Aspartate aminotransferase
BUN: Blood urea nitrogen
CPK: Creatine phosphokinase
DSM-5: Diagnostic and Statistical Manual of Mental Disorders, Fifth Edition
ECG: Electrocardiogram
FAS: Full analysis set
KDSQ-C: Korean Dementia Screening Questionnaire-Cognition
ISI-K: Insomnia Severity Index-Korean
ITT: Intention-to-treat
LDH: Lactate dehydrogenase
LMM: Linear mixed model
PP: Per protocol
PSQI-K: Pittsburgh Sleep Quality Index-Korean
RM ANOVA: Repeated measures ANOVA
SCD-Q: Subjective Cognitive Decline Questionnaire
SF-36: 36-item MOS Short Form Survey
S-GDS: Short version of Geriatric Depression Scale
SNSB: Seoul Neuropsychological Screening Battery
TSH: Thyroid stimulating hormone
